# Author Correction: Iontronic pressure sensor with high sensitivity over ultra-broad linear range enabled by laser-induced gradient micro-pyramids

**DOI:** 10.1038/s41467-023-39675-z

**Published:** 2023-07-03

**Authors:** Ruoxi Yang, Ankan Dutta, Bowen Li, Naveen Tiwari, Wanqing Zhang, Zhenyuan Niu, Yuyan Gao, Daniel Erdely, Xin Xin, Tiejun Li, Huanyu Cheng

**Affiliations:** 1 School of Mechanical Engineering, Hebei University of Technology, 300401 Tianjin, China; 2 Department of Engineering Science and Mechanics, The Pennsylvania State University, University Park, PA 16802 USA; 3 School of Mechanical Engineering, Hebei University of Science & Technology, 050018 Shijiazhuang, China

**Keywords:** Sensors and biosensors, Mechanical engineering, Electrical and electronic engineering

Correction to: *Nature Communications* 10.1038/s41467-023-38274-2, published online 01 June 2023

The original version of this Article contained an error in Fig. 2g, in which the legend of curve ‘GPML_700_ 20 wt%’ was incorrectly swapped with ‘GPML_700_ 50 wt%’, and ‘GPML_500_ 20 wt%’ was incorrectly swapped with ‘GPML_500_ 50 wt%’.

The original version of this Article omitted that ‘(3-Aminopropyl)triethoxysilane (APTES)’ was obtained from Sigma-Aldrich. This has now been included in the ‘Materials and chemicals’ subsection of the ‘Methods’ section.

Finally, the original version of the Supplementary Information contained errors or misplaced information in Supplementary Tables 2, 4, 6. These were as follows:

In Supplementary Table 2

- The “Type”, “Linear Sensing Range (kPa)” and “Linear Sensing Factor (S_p_)” of the pressure sensor reported in previous Supplementary Reference [18], Zhou H, et al. Capacitive pressure sensors containing reliefs on solution-processable hydrogel electrodes. ACS Applied Materials & Interfaces 13, 1441-1451 (2021), have been incorrectly given as “EDL: mold-based”, “7.4”, and “56.98”, respectively. The correct values are “Conventional: mold-based”, “0.86”, and “6.622”. The line, which was previously in the 3^rd^ row, has been moved to the 11^th^ row and is now Supplementary Reference [26].
- The “Structure Fabrication Method” of the pressure sensor reported in Supplementary Reference [29], Yang J, et al. Flexible, Tunable, and Ultrasensitive Capacitive Pressure Sensor with Microconformal Graphene Electrodes. Acs Applied Materials & Interfaces 11, 14997-15006 (2019), has been incorrectly given as “Photolithography Silicon wafer”. The correct value is “UV lithography copper foil”.
- The “Linear Sensing Factor (S_p_)” of the pressure sensor reported in Supplementary Reference [32], Qiu J, et al. Rapid-response, low detection limit, and high-sensitivity capacitive flexible tactile sensor based on three-dimensional porous dielectric layer for wearable electronic skin. ACS applied materials & interfaces 11, 40716-40725 (2019), has been incorrectly given as “18.6”. The correct value is “0.0186”.
- The pressure sensor reported in previous Supplementary Reference [35], Zhang Y, et al. Flexible and Highly Sensitive Pressure Sensors with Surface Discrete Microdomes Made from Self‐Assembled Polymer Microspheres Array. Macromol Chem Phys 221, 2000073 (2020), was incorrectly included in this table. The reported sensor in this work is piezoresistive instead of capacitive. Therefore, this reference has been removed.

In Supplementary Table 4, the 6^th^ row was incorrectly referenced with Supplementary Reference [3]. The correct reference for this row is Supplementary Reference [2], Ji B, et al. Gradient Architecture-Enabled Capacitive Tactile Sensor with High Sensitivity and Ultrabroad Linearity Range. Small 17, e2103312 (2021).

In Supplementary Table 6, the 16^th^ row “Type”, “Sensing Range (kPa)”, and “Sensitivity (kPa-1)” were incorrectly given as “EDL”, “0-7.4”, and “7.7”, respectively. The correct values are “parallel plate”, “0-0.86/0.86-4.90/4.90-7.4”, and “7.7/3.95/1.26”, respectively.

The Supplementary References have been updated to reflect the change of previous Supplementary Reference [18] to Supplementary Reference [26], and removal of previous Supplementary Reference [35].

These have been corrected in both the PDF and HTML versions of the Article. The HTML has also been updated to include a corrected version of the Supplementary Information.

## Supplementary information

Updated Supplementary Information

