# Supplementary material for: Iontronic pressure sensor with high sensitivity over ultra-broad linear range enabled by laser-induced gradient micro-pyramids
Source: Nat Commun. 2023 Jun 1;14:2907. doi: 10.1038/s41467-023-38274-2 (PMC10235028; doi:10.1038/s41467-023-38274-2)
Supplement: Supplementary file 1 — Supplementary Information [file 41467_2023_38274_MOESM1_ESM.pdf]

Supplementary Information

Iontronic pressure sensor with high sensitivity over ultra-broad linear range enabled by laser-induced gradient micro-pyramids

Ruoxi Yang[1, 2], Ankan Dutta[2], Bowen Li[2], Naveen Tiwari[2], Wanqing Zhang[2], Zhenyuan Niu[2], Yuyan Gao[2], Daniel Erdely[2], Xin Xin[2], Tiejun Li[1, 3*], Huanyu Cheng[2*]

[1]School of Mechanical Engineering, Hebei University of Technology, Tianjin, 300401, China

[2]Department of Engineering Science and Mechanics, The Pennsylvania State University, University Park, 16802, USA

[3]School of Mechanical Engineering, Hebei University of Science & Technology, Shijiazhuang, 050018, China

[*]To whom correspondence should be addressed: E-mail: 001036@hebust.edu.cn and huanyu.cheng@psu.edu

This Supplementary Information contains three Supplementary Notes, twelve Supplementary Figures, and six Supplementary Tables.

Note 1. Design of micro-pyramids

The pyramid structures with excessive height (e.g., over 1 mm) can be difficult to encapsulate or package. Buckling may also occur for those with a high aspect ratio as discussed in the "Gradient pyramidal microstructures from laser-ablated molds" Section and Supplementary Figure 3b. The pressure-sensing range is directly relevant to the deformation of the microstructure. In general, the microstructures with high aspect ratios (or larger size) are beneficial for the increased sensitivity [1] (or increased sensing range). As reported in the literature, the pressure sensors with a microstructure size smaller than 100 μm often show a sensing range of less than 50 kPa (Supplementary Table 4). In contrast, the sensors with a large microstructure size (e.g., side length bigger than 500 μm) can provide a sensing range of more than 1000 kPa[2, 3]. As for the pyramidal microstructures, when the L/H ratio (L is the bottom side length and H is the height) is $\sqrt{2}$, the sensors can exhibit a balanced performance between sensitivity and linearity [4, 5, 6].

To imitate pyramid structures and also avoid buckling, we design the gradient structure $GPML_{700}$ structure with L/H of 1.2 and 2.2, which resulted in linear sensing ranges (for all three ionic liquid concentrations).

Note 2. Theoretical analysis of UPM and GPM at the dielectric/electrode interface to understand the enhanced sensitivity without bending

The normalized cross-section w of the contact surface of the pyramid microstructure increases proportionally to the square root of the compression force F against the pyramid[7].

$$w \propto \sqrt{F}. \tag{1}$$

However, the capacitance C is directly proportional to the area or square of the cross-section of the contact surface, thus, the capacitance becomes directly proportional to the compressive force.

$$C \propto w^2 \propto F \tag{2}$$

The linear dependence of capacitance and force thus originates from the non-linear relationship between the cross-section and compressive force given in equation (1). For gradient microstructure, the effective cross-section w_{eff} of the contact increases in a cascading order for each new pillar $i \leq N$ after exceeding every corresponding force π_i. The corresponding force π_i depends on the gradient of the microstructure, as the gradient increases, more exceeding force π_i is required to start deformation of the ith pillar. For example, the corresponding force will be zero for all the pillars for a uniform pillar distribution, whereas, for a gradient pillar distribution, only the exceeding force π_1 corresponding to the initially deformed pillar $i = 1$ will be zero, and this force increases with the pillar index $\pi_i < \pi_{i+1}\ \forall i \leq N$. The force F_i deforms each pillar i with width w_i as the following relationship

$$w_i(F) = \begin{cases} w_i \propto \sqrt{F_i}, & F > \pi_i \\ 0, & F \leq \pi_i \end{cases}$$

The effective cross-section w_{eff} is given by

$$w_{\text{eff}} = \sum_{i=1}^{N(F)} w_i(F) \propto \sum_{i=1}^{N(F)} \sqrt{F_i}. \tag{3}$$

Using Cauchy–Schwarz inequality,

$$w_{\text{eff}} \propto \sum_{i=1}^{N(F)} \sqrt{F_i} \leq \sqrt{\sum_{i=1}^{N(F)} F_i} \approx \sqrt{N(F)\langle F\rangle}. \tag{4}$$

Thereby, the effective capacitance C_{eff} is given by

$$C_{\text{eff}} \leq N(F)\langle F\rangle, \tag{5}$$

Where $N(F)$ is the number of pillars deformed with $\pi_N < F < \pi_{N+1}$ and average force $\frac{1}{N(F)}\sum_{i=1}^{N(F)} F_i = \langle F \rangle$. The number of pillars $N(F)$ increases monotonically with force as the force increases with the number of pillars deformed. Moreover, the average force $\langle F \rangle$ corresponds to the pressure applied P over the sensor cross sectional area A. Therefore, the number of pillars $N(F)$ is also a function of pressure applied $N(P)$.

The slope of effective capacitance C_{eff} with pressure or sensitivity S is thus given by

$$S = \frac{\partial C_{\mathrm{eff}}}{\partial P} \leq kAN(P), \quad (6)$$

Where k is the proportional constant. As the effective capacitance depends on the square of the effective cross-section, the slope of the capacitance force is upper-bounded by the number of pillars N that depends on the pressure P.

Note 3. Mathematical model of the electric field distribution

The governing equations for electrostatics in the ionic liquid domain are given as follows:

$$\nabla \cdot D = \rho_v \tag{7}$$

$$D = \varepsilon_0 \det(F)(F^T F)^{-1} E + \varepsilon_0 \chi_r E + \mathrm{P}(\varepsilon_{elastic}) \tag{8}$$

$$E = -\nabla V \tag{9}$$

where ρ_v is the formed free electron surface charge density and *D* is the electric displacement vector. The domain has a deformation gradient F, electric susceptibility of χ_r with a polarization of P in the elastic limit. The domain is under an electric field caused by the potential difference V between the boundaries of the domain. The governing equations for compressible neo-Hookean materials in the mechanical domain are given as follows:

$$0 = \nabla \cdot (FS)^T + F_v \tag{10}$$

$$S = \frac{\partial W_s}{\partial \epsilon} \tag{11}$$

$$\epsilon = \frac{1}{2}(F^T F - I) \tag{12}$$

$$W_s = \frac{1}{2}\mu(I_1 - 3) - \mu \log(J_{elastic}) + \frac{1}{2}\lambda \log(J_{elastic})^2 \tag{13}$$

where W_s is the elastic strain energy density that is a function of elastic strain state ϵ. For a compressible neo-Hookean material, the elastic strain energy depends on the elastic volume ratio $J_{elastic}$, Lame parameters μ, λ, and the first invariant of the elastic right Cauchy-Green deformation tensor I_1. The governing equations for the transport of dilute species in the ionic liquid domain are given as follows:

$$\nabla \cdot J = R \tag{14}$$

$$J = -D\nabla c - z u_m F_a c \nabla V \tag{15}$$

where the concentration c of each species and diffusion constant *D* contribute to the current J. The other contribution comes from the migration of species of charge z and mobility u_m due to the electric potential *V*. The space charge coupling between electrostatics and the transport of dilute species is given by the following governing equations:

$$\nabla \cdot D = \rho_v \tag{16}$$

$$\rho_v = F_a \sum_i z_i c_i \tag{17}$$

where F_a is Faraday's constant.

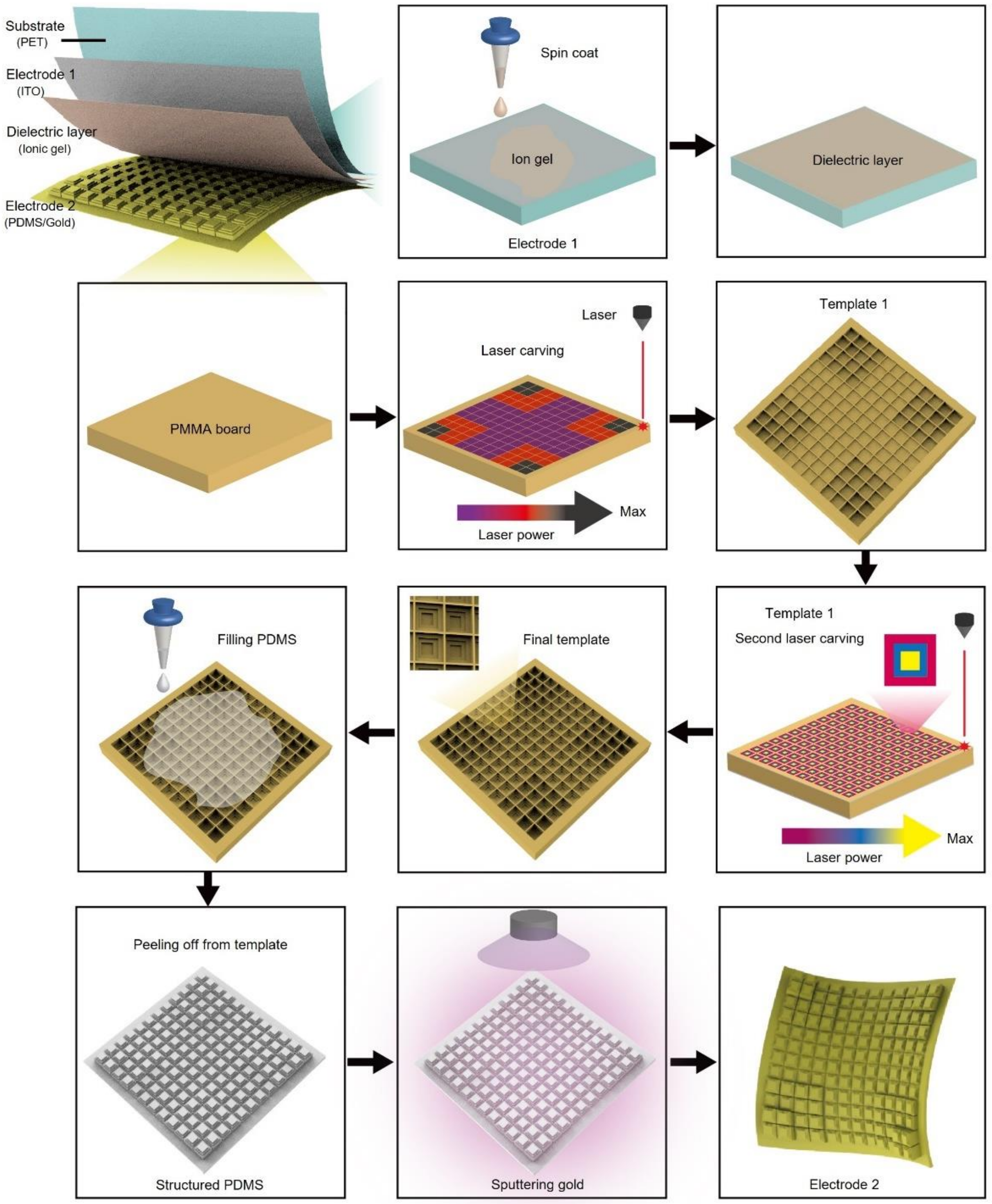

Supplementary Fig. 1. Schematic showing the fabrication process of the iontronic pressure sensor.

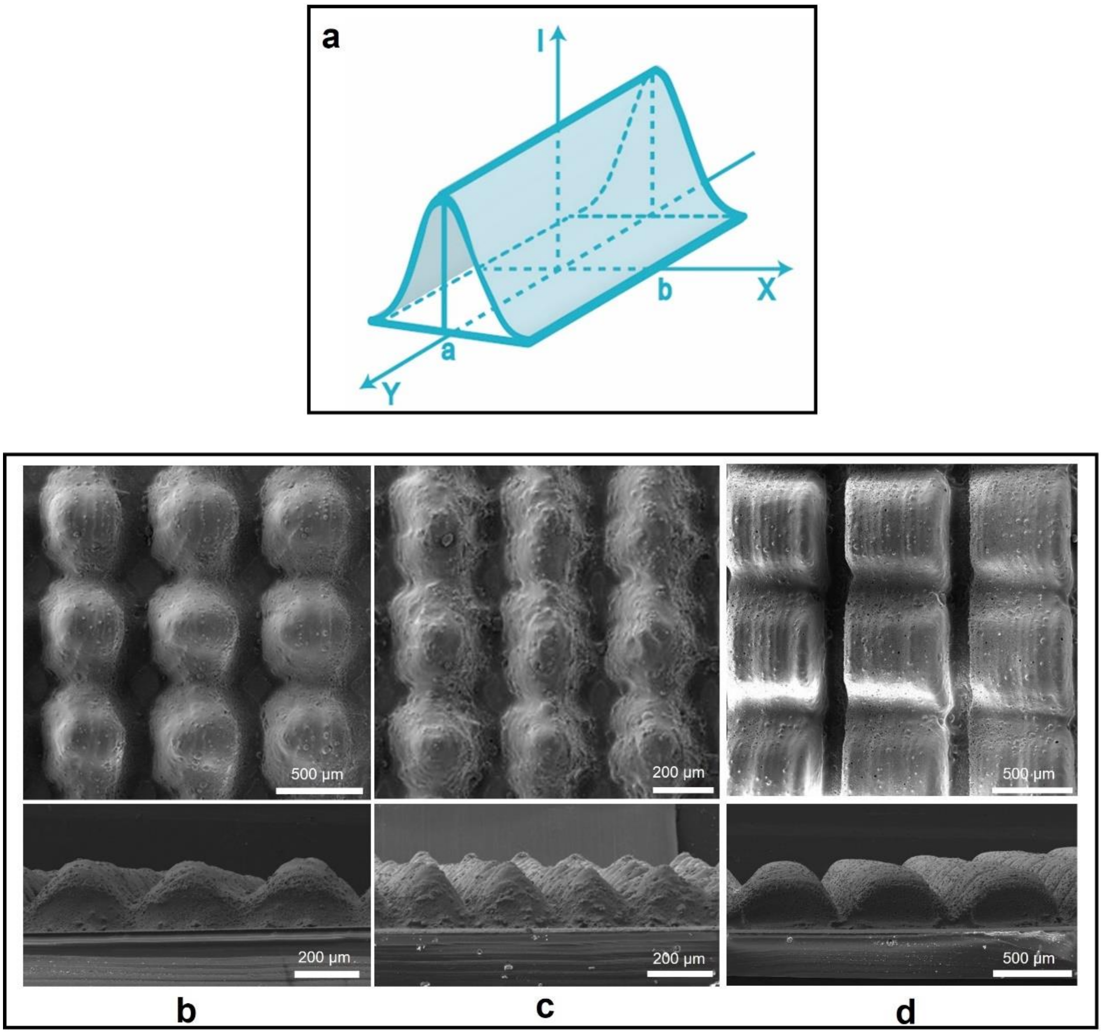

Supplementary Fig. 2. Different microstructures created by the laser with a Gaussian beam. **a** Schematic showing the laser power distribution (I: intensity; Y: running direction). SEM images showing **b** conical frustums, **c** cone, and **d** square frustums microstructures.

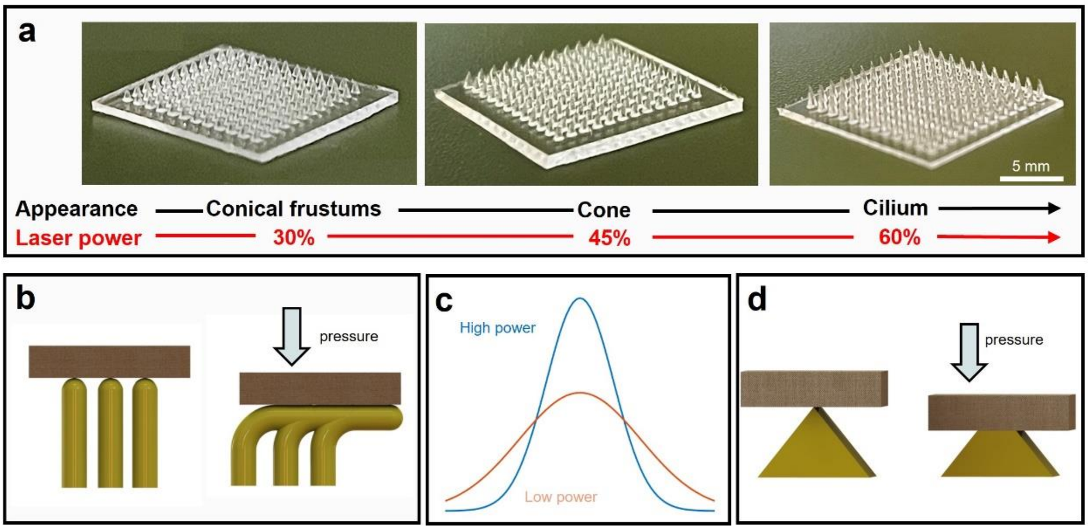

Supplementary Fig. 3. Fabrication of single-layered microstructures. **a** Optical images showing the microstructures with higher aspect ratios as the laser power increases (fabricated with the square pattern). **b** Schematic showing the buckling of the slender microstructures upon pressure loading. **c** Comparison between the high (blue) and low (red) laser power with a Gaussian distribution. **d** Schematic showing the deformation of a square frustum upon pressure loading.

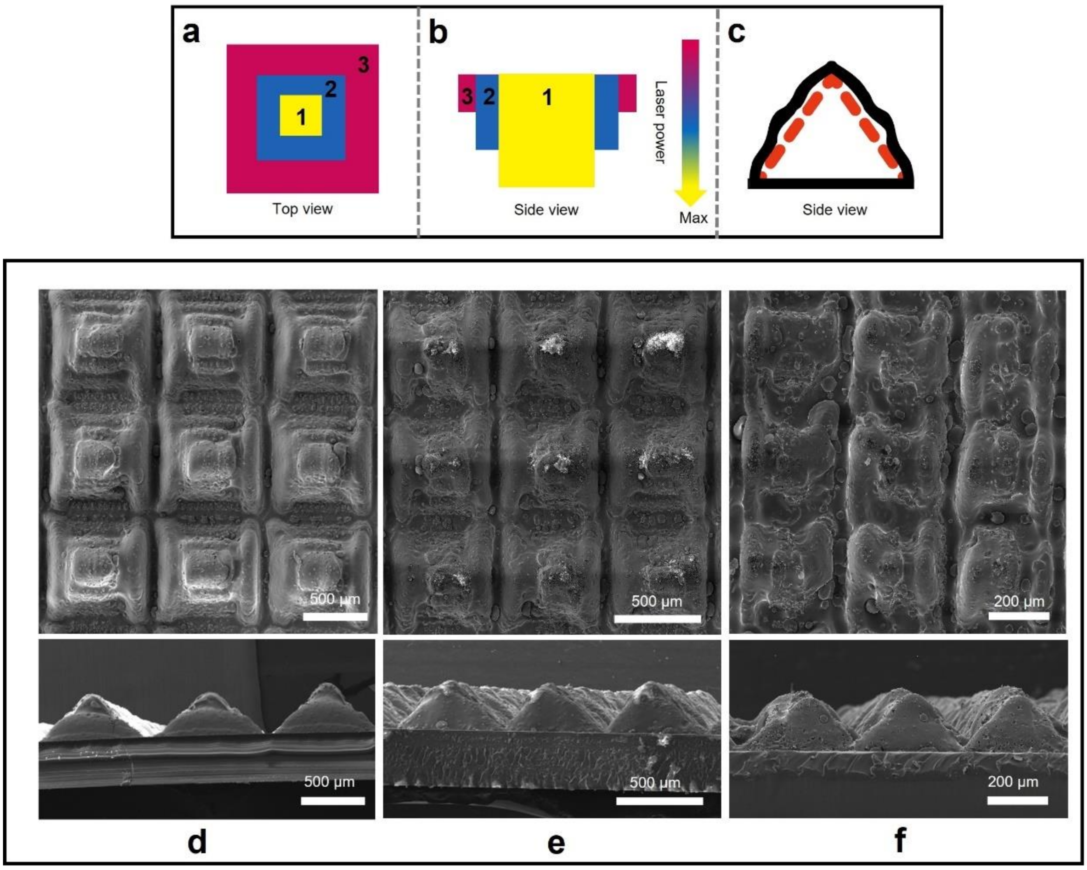

Supplementary Fig. 4. Fabrication of pyramidal microstructures with tri-layered patterns. Schematic showing the **a** top and side **b** views of the laser-ablating patterns for creating the PMMA mold and **c** the resulting PDMS pyramid microstructure (without shape edges because of the Gaussian distribution of the laser power) de-molded from the PMMA mold. Note: the numerical number represents the sequence of the laser ablation. SEM images showing the pyramidal structures with different sizes: **d** 700 µm and **e** 500 µm in the unit cell, as well as **f** the failed pyramid created by three square patterns with a small difference.

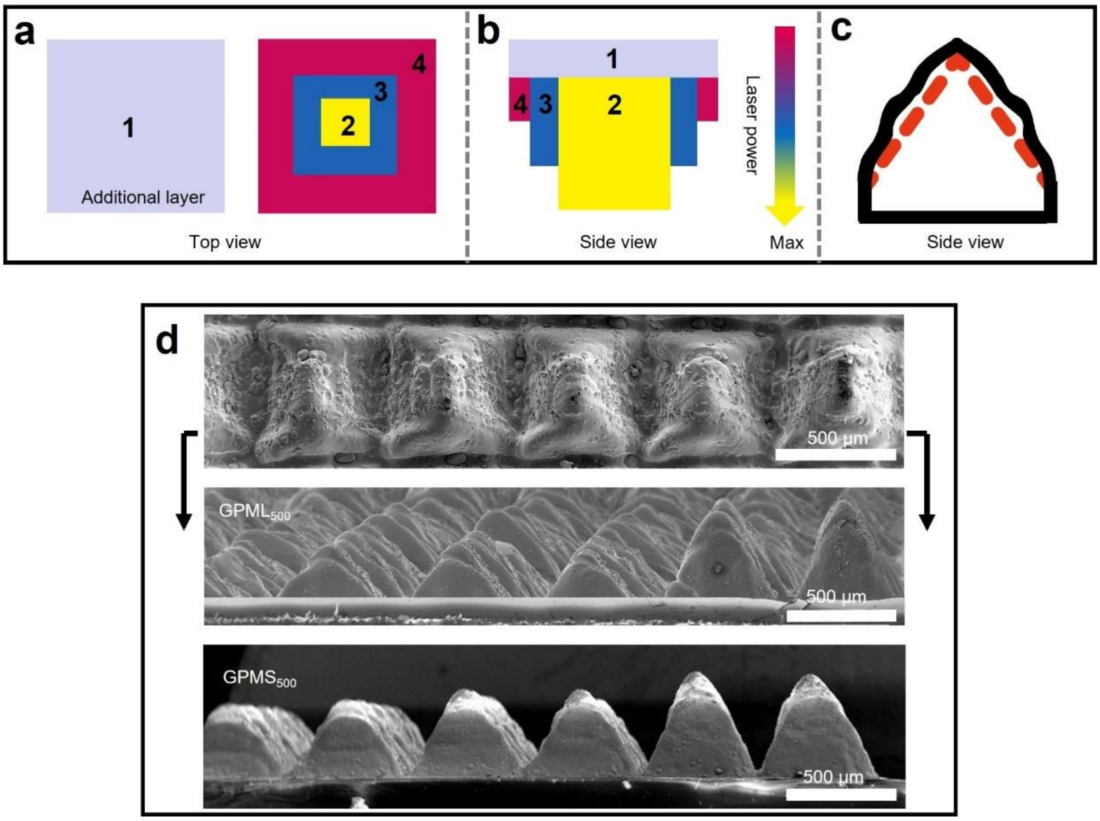

Supplementary Fig. 5. Fabrication of gradient pyramidal microstructures with an additional base layer (lilac with a varied power). Schematic showing the **a** top and side **b** views of the laser-ablating patterns for creating the PMMA mold and **c** the resulting PDMS pyramid microstructure. Note: the numerical number represents the sequence of the laser ablation. **d** SEM images showing gradient pyramidal microstructures ($GPML_{500}$) from the top and side views.

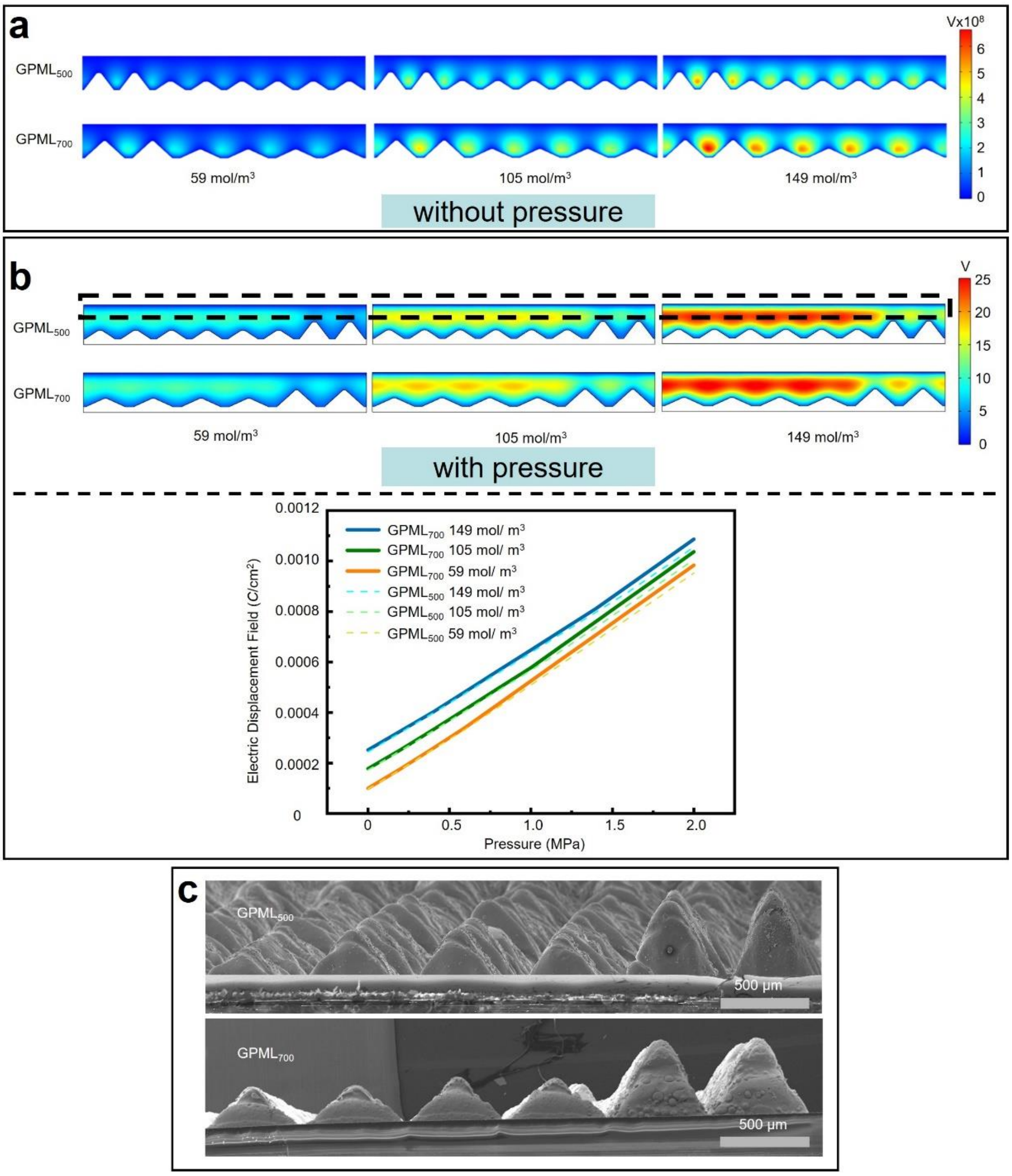

Supplementary Fig. 6. The comparison between $GPML_{500}$ and $GPML_{700}$ in terms of electric potential distributions and microstructures. Nonuniform electric potential distribution in $GPML_{500}$ and $GPML_{700}$ **a** before and **b** after pressure loading. The change in the electric displacement field with the increasing pressure for varying IL concentrations is shown at the bottom (the top boundary was selected as the probe). **c** Side-view SEM images showing the difference in the size of pyramids between $GPML_{500}$ and $GPML_{700}$.

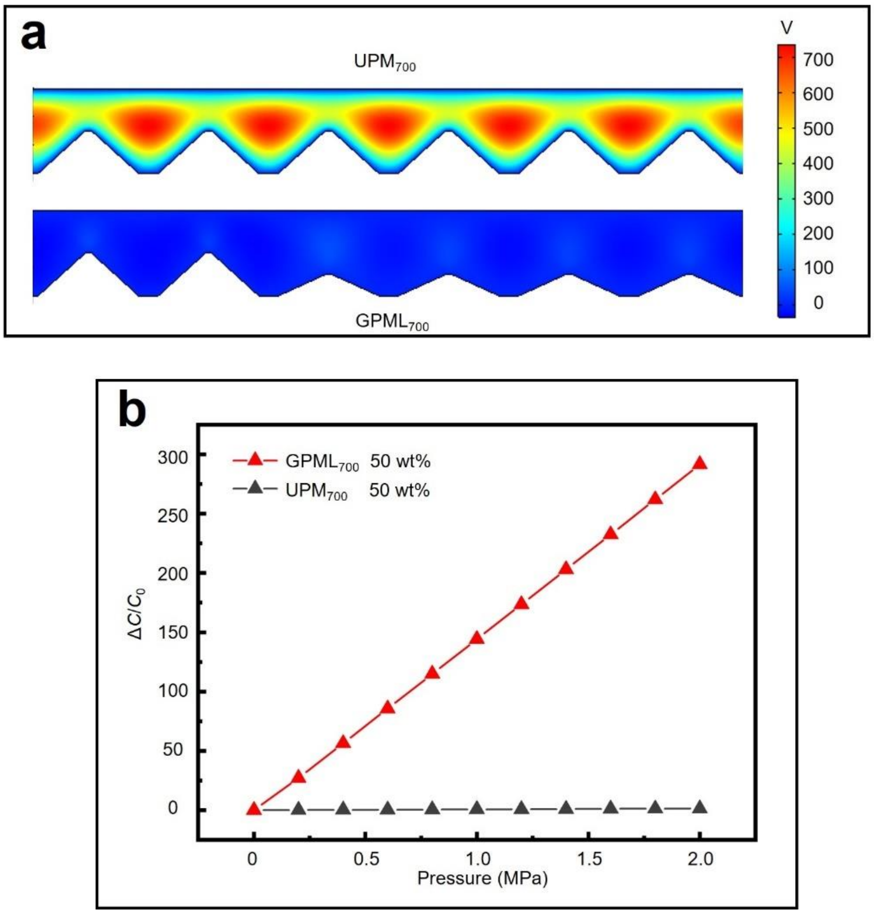

Supplementary Fig. 7. The comparison between UPM_{700} and $GPML_{700}$ in terms of the electric potential distribution and calculated sensitivity. **a** Comparison in the potential distribution between the uniform (top) and gradient (bottom) structures. **b** The normalized relative capacitance changes as a function of the applied pressure between the gradient and uniform structures, with a 220-fold increase in the sensitivity for the gradient structure.

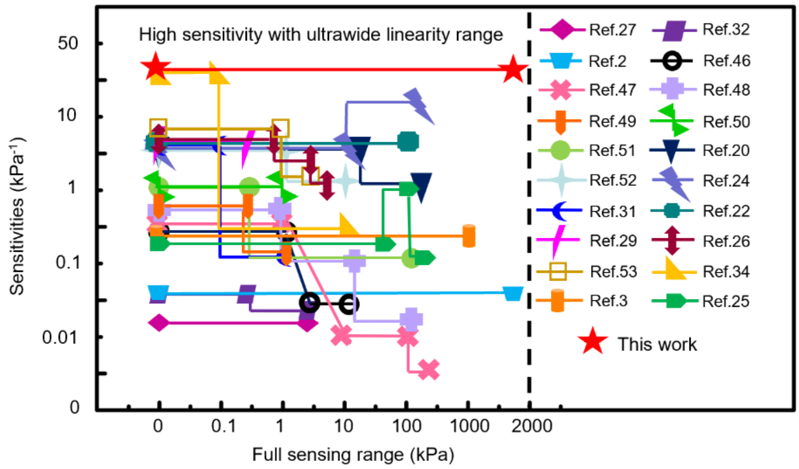

Supplementary Fig. 8. Performance comparison in the sensitivity and full sensing range between the flexible iontronic pressure sensor from this work and other capacitive pressure sensors. Note: The reference numbers correspond to those in Supplementary Table 6.

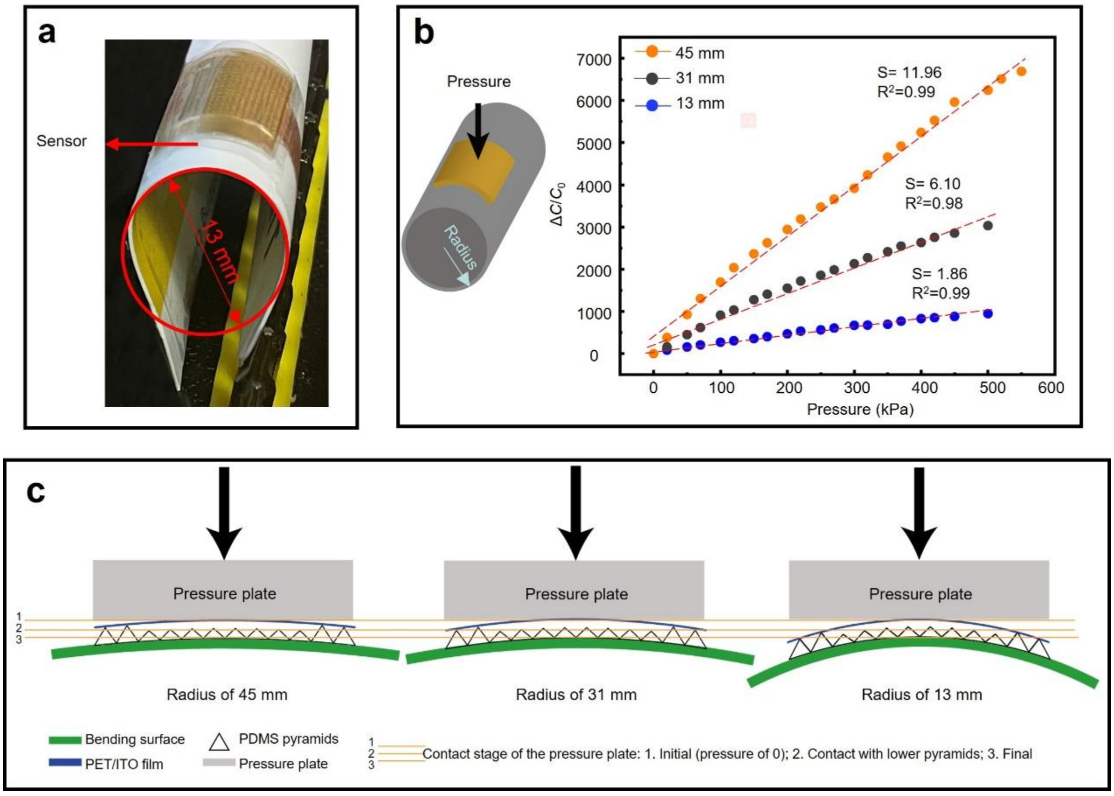

Supplementary Fig. 9. Demonstration of the flexible sensor and its performance under bending. **a** Optical image of the iontronic sensor bent over a diameter of 13 mm. **b** The pressure sensing performance and **c** schematic diagram of the iontronic pressure sensor under different bending conditions.

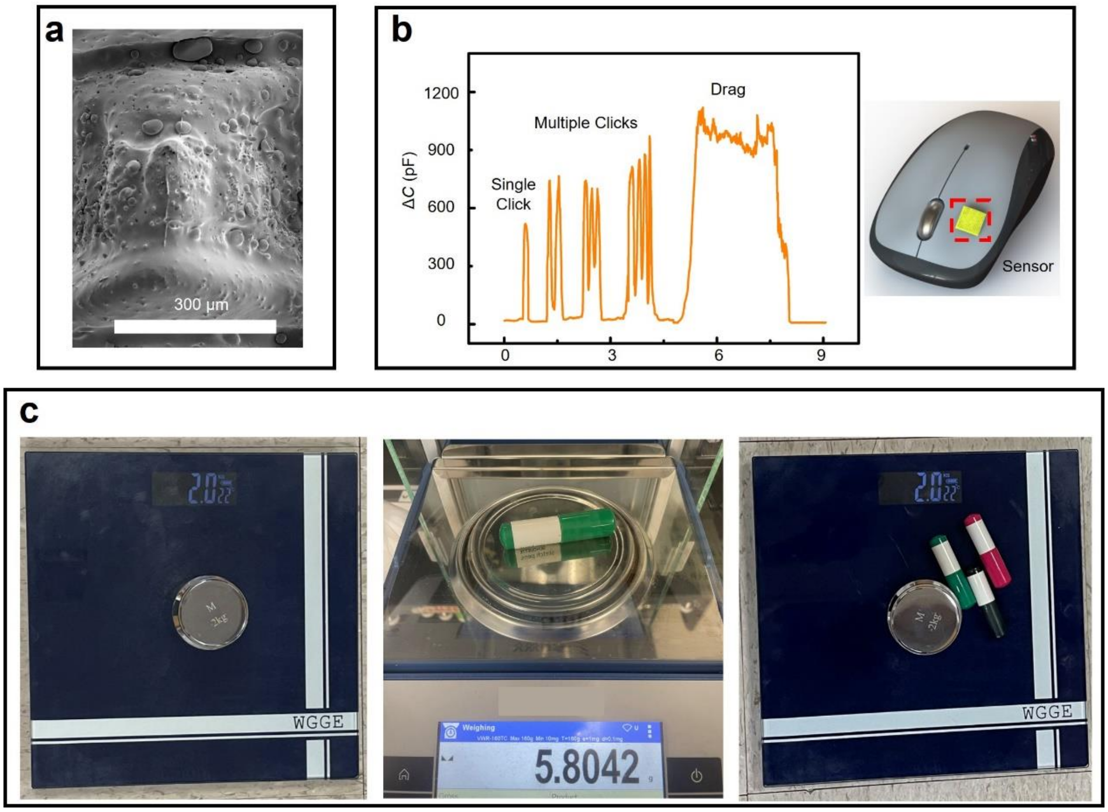

Supplementary Fig. 10. Tip of the micropyramid, response from the sensor on the mouse, and pressure measurement using a commercial weighing scale. **a** Top-view SEM image showing the rough surface on the tip of a single pyramidal microstructure. **b** Measurement of mouse movements for static and dynamic pressure detection, including single/double/triple clicks and hold-and-drag operation (IL of 35 wt%). **c** Calculation of the pressure resolution of the commercial weight scale with pens.

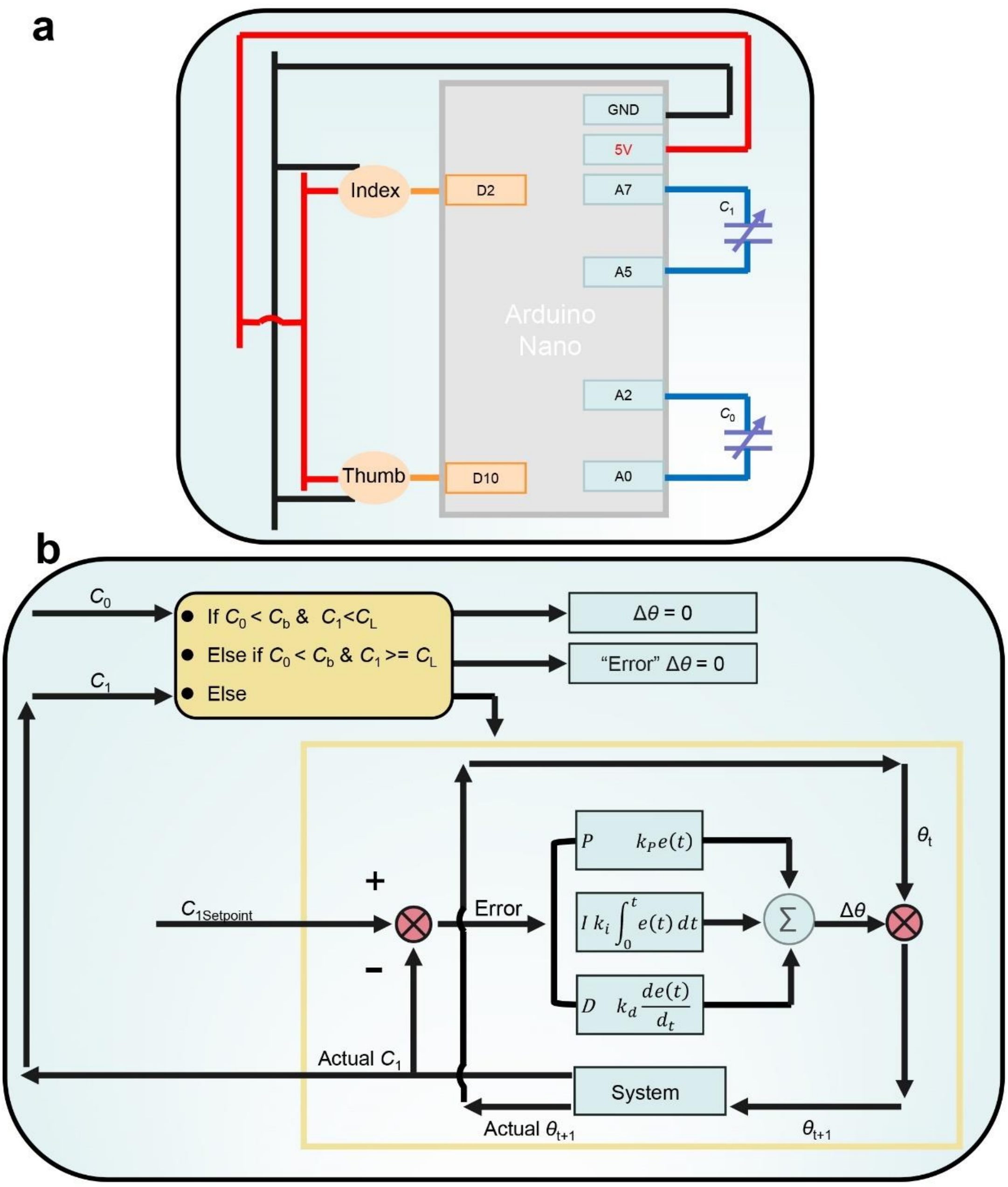

Supplementary Fig. 11. The operating mechanism of the robotic hand. **a** Circuit diagram of the robotic hand with two iontronic pressure sensors. **b** Schematic diagram showing the use of the proportion-integration-differentiation (PID) system to control the robotic hand based on the capacitance measurements C_0/C_1 from the iontronic pressure sensor on the thumb/index finger. C_b and C_L are the boot thresholds of the sensor on the thumb and index finger, respectively. θ is the rotation of the motor for the robotic finger with θ_t for the present value. $C_{1setpoint}$ is the desired value and its difference with C_1 is noted as error.

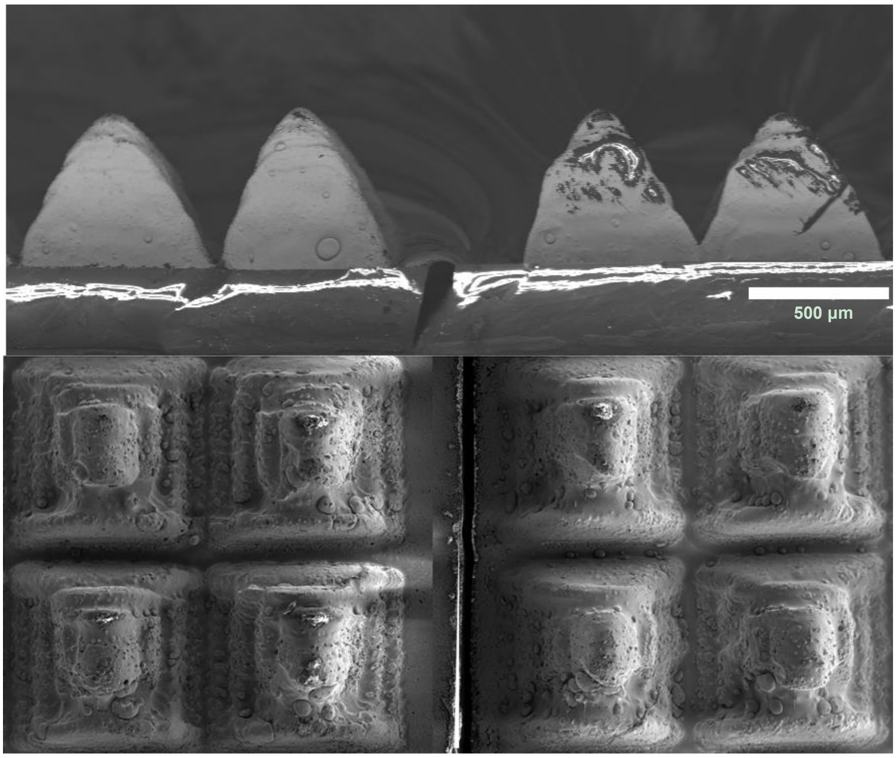

Supplementary Fig. 12. The comparison between microstructures obtained from two PMMA templates that were separately created by using the same laser parameters, demonstrating reasonably good consistency in the morphology (e.g., height, outline, and surface topography).

Supplementary Table 1. Different fabrication methods comparison for microstructure templates

Method	Materials	Facilities	Fabrication process	Environment requirement	Notes
Photolithography silica template[8, 9]	1. Silicon wafer<br>2. Photoresist<br>3. Silicon nitride<br>4. Potassium hydroxide<br>5. DI water<br>6. Trichloro-(1H,1H,2H,2H-perfluorooctyl) silane<br>7. IPA<br>8. Acetone	1. Cleanroom<br>2. Lithography equipment<br>3. Spin coater<br>4. Wet bench<br>5. O_2 plasma	1. Silicon nitride deposition<br>2. Mask preparation<br>3. Silicon etching<br>4. Remove the remaining silicon oxide<br>5. O_2 plasma treatment<br>6. Trichloro-(1H,1H,2H,2H-perfluorooctyl) silane deposition	High temperature for etching	1. Templates are regular, uniform, and high precision, along with complex fabrication processes.<br>2. High equipment requirements.<br>3. The template fabrication time > 5 h
3D printing[10, 11]	Special materials for 3D printers (e.g., ABS, PLA,	3D printer	Printing (3D modeling needed)	Ambient conditions	1. Additive manufacture (low throughput)

	etc., depending on the type of printers)				2. Some materials (e.g., PLA) can not bear high temperatures (e.g., more than 40℃)[10], taking longer time to cure polymer.
Electrochemical oxidation aluminum oxide template[12, 13, 14]	1. Aluminum plates<br>2. Perchloride acid<br>3. Ethanol<br>4. Oxalic acid<br>5. Phosphoric acid<br>6. Chromic acid<br>7. Trimethylchlorosilane gas	1. Voltage supply device<br>2. Temperature control device	1. Annealing<br>2. Electrochemical polishing<br>3. Two-step anodization<br>4. Surface treatment	1. High temperature (annealing and chemical etching)<br>2. Low temperature (anodization)<br>3. DC voltage	1. Structure limitation (most of them are templates for cone and column).<br>2. High density and uniform parallel nanopores.
Micro-engraving[2, 15]	Plastic boards (e.g., polycarbonate)	Engraving machine and microdrill	1. Cutter switching<br>2. Engraving	Ambient conditions	1. The shape and size of microstructures are limited by cutters.<br>2. Cutters are easily getting abrased.
Laser ablation	PMMA boards	CO_2 laser system	Printing	Ambient conditions	1. Easy to start.<br>2. High prototyping efficiency.

					3. High design flexibility to adjust the size/shape/height/density of microstructures.

Supplementary Table 2. Performance comparison of different capacitive pressure sensors

	Number	Type	Structure fabrication method	Sensitivity (kPa^{-1})	Linear Sensing Range (kPa)	Linear sensing factor (S_P)	Pressure resolution	Response/recovery time (ms)	LOD (Pa)	Ref.
1	1	EDL: mold-based	Photolithography Silicon wafer	1.3	3	3.9	0.02% (base pressure of 5 kPa)	15/15	0.2	[16]
	2	EDL: mold-based	Photolithography Silicon wafer	7.49	6	44.94	NR	9/9	0.9	[17]
	3	EDL: mold-based	3D print mold	49.1	4-485	2.37×10^4	NR	0.61/3.63	NR	[18]
2	4	EDL: structure-transfer	Transfer sandpaper structure	3302	10	3.302×10^4	0.0056% (base pressure of 320 kPa)	9/18	0.08	[19]
	5	EDL: structure-transfer	Transfer sandpaper structure	5.5	30	165	NR	70.4/92.8	2	[20]
	6	EDL: structure-	Transfer sandpaper	9.17	0.013-2063	1.89×10^4	NR	5/16	13	[21]

		transfer	structure							
	7	EDL: structure-transfer	Transfer sandpaper structure	6.94	100	694	NR	48/	2.88	[22]
	8	EDL: structure-transfer	Transfer sandpaper structure	200	60	1.2×10^4	1.4% (base weight of 71 kg)	98/70	20	[23]
	9	EDL: structure-transfer	Directly using fabric materials	6.5	10	65	NR	30/30	7.5	[24]
	10	EDL: structure-transfer	Directly using fabric materials	0.24	70	16.8	NR	18	35	[25]
3	11	Conventional: mold-based	Micro-engraving plastic template	7.7	0.86	6.622	NR	NR	NR	[26]
	12	Conventional: mold-based	Photolithography Silicon wafer	2.51	10	25.1	NR	84/117	2	[1]
	13	Conventional: mold-based	Photolithography Silicon wafer	0.022	5	0.11	NR	NR	NR	[27]
	14	Conventional: mold-based	Photolithography Silicon wafer	0.43	1	0.43	NR	33/33	3.4	[28]
	15	Conventional: mold-based	UV lithography copper foil	7.68	0.5	3.84	NR	30/28	1 mg	[29]
	16	Conventional: mold-based	Commercially anodic aluminum oxide template	0.35	2	0.7	NR	48/60	4	[30]
	17	Conventional: mold-based	Commercially anodic	6.583	0.1	0.6583	NR	48/36	3	[31]

			aluminum oxide template							
	18	Conventional: mold-based	Polyurethane sponge skeletons	0.062	0.3	0.0186	NR	45/83	3	[32]
	19	Conventional: mold-based	Nickel foam template	3.13	1	3.13	NR	94/	0.07	[33]
	20	Conventional: mold-based	Micro-engraving plastic template	0.065	1700	111	1% (base pressure of 1000 kPa)	100/100	/	[2]
4	21	Conventional: structure-transfer	Transfer the outline of polystyrene spheres	30.2	0.13	3.926	NR	25/50	0.7	[34]
	22	Conventional: structure-transfer	Transfer the outline of reed leaves	0.6	1	0.6	NR	180/120	4.5	[35]
	23	Conventional: structure-transfer	Transfer the outline of bamboo leaves	2.08	1	2.08	NR	500/700	20	[36]
	24	Conventional: structure-transfer	Transfer the outline of lotus leaves	1.2	2	2.4	NR	36/58	0.8	[37]
	25	Conventional: structure-transfer	Transfer the surface structure of obscured glass	1.1	0.5	0.55	NR	1000/NR	1	[38]
	26	Conventional: structure-transfer	Directly using dried flower petal as dielectric layer	1.54	1	1.54	NR	NR	0.6	[39]

	27	Conventional: structure-transfer	Transfer the surface structure of paper	0.62	2	1.24	NR	NR	6	[40]
		EDL: mold-based	Laser-ablated PMMA board	33.7	1700	5.729×10^4	0.00725% (base pressure of 2000 kPa)	6/11	0.36	Our work

Note: NR (not reported), $S_P=S\bullet\Delta P$ (S: sensitivity and ΔP: the corresponding linear sensing range)

Supplementary Table 3. Different designs in the laser ablation pattern for varying microstructures

Number	Pattern	Laser power (color sequence)	Side length/diameter (μm)	Distribution
a (S2-b)	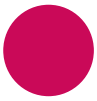	30%	500	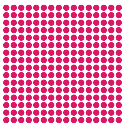
b (S2-c)	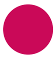	30%	250	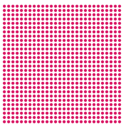
c (S2-d)	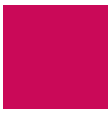	30%	700	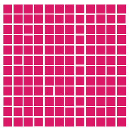
d (S4-d)	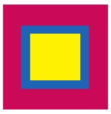	30%/25%/20% yellow/blue/pink	704/563/422	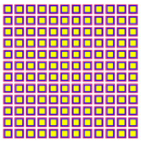
e (S4-e)	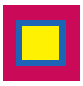	30%/25%/20% yellow/blue/pink	526/421/316	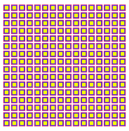
f (S4-f)	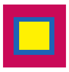	30%/25%/20% yellow/blue/pink	352/282/211	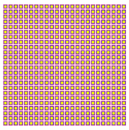
GPMS$_{500}$	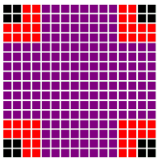	25%/21%/14% black/red/purple	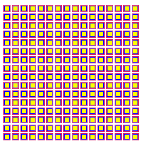	30%/25%/20% yellow/blue/pink
GPML$_{500}$	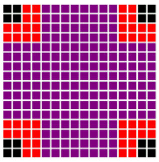	25%/10%/10% black/red/purple	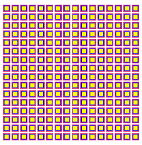	30%/25%/20% yellow/blue/pink
GPMS$_{700}$	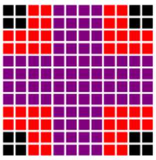	25%/21%/14% black/red/purple	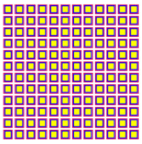	30%/25%/20% yellow/blue/pink
GPML$_{700}$	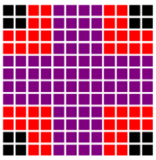	25%/10%/10% black/red/purple	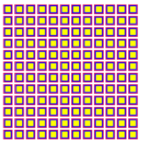	30%/25%/20% yellow/blue/pink

Supplementary Table 4. Microstructures with different aspect ratios used in the pressure sensors

Principle	Structure	Side length (L) (μm)	Height (H) (μm)	L/H	Maximum Sensing range (kPa)	Ref.
Piezoresistant	Pyramid	4.64	2.97	1.56	3	41
Conventional capacitor	Pyramid	50	30.25	1.65	35	42
Conventional capacitor	Pyramid	4.88	1.65	2.97	7	43
EDL-based capacitor	Pyramid	6.49	3.5	1.85	50	44
Piezoelectric	Pyramid	60	42	1.42	10	45
Conventional capacitor	Gradient Dome	500	700; 450; 200	/	1700	2
EDL-based capacitor	Gradient pyramids	700	570; 310	1.2; 2.2	3000	

Supplementary Table 5. Molarity calculation

wt%	m_{IL}(g)	$m_{PVDF\text{-}HFP}$ (g)	$m_{acetone}$ (g)	Molarity (mol/m^3)
20	0.06	0.3	2	59
35	0.105	0.3	2	105
50	0.15	0.3	2	149

$n_{20\ wt\%}=m_{IL20\ wt\%}/\mathrm{MW}=0.00015$ (mol)

$n_{35\ wt\%}=m_{IL35\ wt\%}/\mathrm{MW}=0.000268$ (mol)

$n_{50\ wt\%}=m_{IL50\ wt\%}/\mathrm{MW}=0.00038$ (mol)

$v=m_{acetone}/\rho_{acetone}=2.55\times10^{-6}$ (m^3)

$Con_{20\ wt\%}=n_{20\ wt\%}/v\approx59$ (mol/m^3)

$Con_{35\ wt\%}=n_{35\ wt\%}/v\approx105$ (mol/m^3)

$Con_{50\ wt\%}=n_{50\ wt\%}/v\approx149$ (mol/m^3)

n: amount of substance of IL; *m*: mass; MW: molar weight of IL; *v*: volume of the acetone; *Con*: molarity; wt%: weight percent of the IL in PVDF-HFP. Density of acetone (ρ): 0.784g/cm^3 and MW=391.3

Supplementary Table 6. Sensing range comparison between this work and other capacitive pressure sensors

Number	Types (EDL/parallel plate)	Sensing range (kPa)	Sensitivities (kPa^{-1})	Material	Structure	Parameters	Ref.
1	parallel plate	0-5	0.022	PDMS	Pyramid (dielectric layer)	(d, ε)	[27]
2	parallel plate	0-0.3	0.062	Graphene nanoplatelets /MWCNTs /Silicone rubber	Porous structure (dielectric layer)	(d, ε)	[32]
		0.3-4.5	0.033				
3	parallel plate	0-1700	0.065	CNT/PDMS	Different height domes (dielectric layer)	(s)	[2]
4	parallel plate	0-1.5	0.42	PDMS	Tilted micropillar (dielectric layer)	(d, ε)	[46]
		5-14	0.04				
5	parallel plate	0-1	0.51	$Ti_3C_2T_x$/(PVDF-TrFE) composite	Nanofibrous scaffolds (dielectric layer)	(d, ε)	[47]
		10-150	0.01				
		150-400	0.006				
6	parallel plate	0-1.6	0.73	PU and calcium copper titanate (high permittivity)	Sponge (dielectric layer)	(d, ε)	[48]
		1.6-22.8	0.135				
		22.8-120	0.026				
7	parallel plate	0-0.5	0.854	Boron Nitride/PDMS	Foam (dielectric layer)	(d, ε)	[49]
		0.55-2.1	0.29				
8	parallel	0-1	1.12	PVDF and insulating PMMA	PVDF Nanofiber	(d, ε)	[50]

	plate				and PMMA microbeads (dielectric layer)		
9	EDL	0-0.5<br>0.5-120	1.194<br>0.109	PVDF and [BMIM]PF_6	Porous PVDF with [BMIM]PF_6 (dielectric layer)	(*s*)	51
10	EDL	0-30<br>30-250	5.5<br>1.5	PVA and a superhydrophilic bis(trifluoromethane)sulfonimide lithium salt (LS), and $Ti_3C_2T_x$	Copy sandpaper structure (electrodes)	(*s*)	20
11	parallel plate	0-2<br>2-10	6.42<br>1.72	Ecoflex−MWCNTs	Porous (dielectric layer)	(d, ε)	52
12	EDL	0-10<br>10-175	6.5<br>13.5	[BMIM]·PF_6	Fabric (dielectric layer)	(*s*)	24
13	parallel plate	0-0.1<br>0.1-1	6.583<br>0.125	P(VDF-TrFE) and DMF	Interlocked asymmetric-nanocones (dielectric layer)	(d, ε)	31
14	EDL	0-100	6.94	(PVDF-HFP) and ([EMI][TFSA])	Copy sandpaper structure (dielectric layer)	(s)	22
15	parallel plate	0-0.5	7.68	Graphene	Three-dimensional microconformal	(d, ε)	29

					graphene (electrode)		
16	parallel plate	0-0.86	7.7	PVA/PANI	Cone (electrode)	(s)	[26]
		0.86-4.90	3.95				
		4.90-7.4	1.26				
17	parallel plate	0-1	8.31	TPU-dielectric layer TPU and AgNW-electrodes	Fabric (dielectric layer)	(d, ε, s)	[53]
		1-5	2.32				
18	parallel plate	0-0.13	30.2	PVDF (dielectric layer) and gold (electrode)	Dome (electrode)	(d, s)	[34]
		0.13-10	0.47				
19	parallel plate	1000	0.314	CIP/NdFeB/PDMS and CNT/PDMS	Cilia array and dome array	(ε, s, d)	[3]
20	EDL	0-70	0.24	Polyacrylamide-Nacl hydrogel	Fabric electrodes	(s)	[25]
		70-150	1.5				
		150-330	0.13				
	EDL	1700	33.7	P(VDF-HFP) and ([EMI][TFSI])	Gradient pyramids	(s)	Our work

Note: d, ε, and s represent the distance between two electrodes, dielectric constant, and contact area between electrode and dielectric layer, respectively.

Supplementary References:

1. Zhang Z*, et al.* Highly sensitive capacitive pressure sensor based on a micropyramid array for health and motion monitoring. *Advanced Electronic Materials* **7**, 2100174 (2021).

2. Ji B*, et al.* Gradient Architecture-Enabled Capacitive Tactile Sensor with High Sensitivity and Ultrabroad Linearity Range. *Small* **17**, e2103312 (2021).

3. Ji B, Zhou Q, Hu B, Zhong J, Zhou J, Zhou B. Bio-inspired hybrid dielectric for capacitive and triboelectric tactile sensors with high sensitivity and ultrawide linearity range. *Adv Mater* **33**, 2100859 (2021).

4. Deng W*, et al.* Microstructure-based interfacial tuning mechanism of capacitive pressure sensors for electronic skin. *Journal of Sensors* **2016**, (2016).

5. Tee BCK, Chortos A, Dunn RR, Schwartz G, Eason E, Bao Z. Tunable flexible pressure sensors using microstructured elastomer geometries for intuitive electronics. *Adv Funct Mater* **24**, 5427-5434 (2014).

6. He B, Yan Z, Zhou Y, Zhou J, Wang Q, Wang Z. FEM and experimental studies of flexible pressure sensors with micro-structured dielectric layers. *J Micromech Microeng* **28**, 105001 (2018).

7. Liu S, Olvera de la Cruz M. Deformation of elastomeric pyramid pen arrays in cantilever-free scanning probe lithography. *Journal of Polymer Science Part B: Polymer Physics* **56**, 731-738 (2018).

8. Lee S*, et al.* A highly sensitive bending sensor based on controlled crack formation integrated with an energy harvesting pyramid layer. *Advanced Materials Technologies* **3**, 1800307 (2018).

9. Jung M*, et al.* Transparent and flexible mayan-pyramid-based pressure sensor using facile-transferred indium tin oxide for bimodal sensor applications. *Scientific reports* **9**, 14040 (2019).

10. Kang B, Hyeon J, So H. Facile microfabrication of 3-dimensional (3D) hydrophobic polymer surfaces using 3D printing technology. *Appl Surf Sci* **499**, 143733 (2020).

11. Zhuo B, Chen S, Zhao M, Guo X. High sensitivity flexible capacitive pressure sensor using polydimethylsiloxane elastomer dielectric layer micro-structured by 3-D printed mold. *IEEE Journal of the Electron Devices Society* **5**, 219-223 (2017).

12. Hoang XT, Nguyen DT, Dong BC, Nguyen HN. Fabrication of carbon nanostructures from polymeric precursor by using anodic aluminum oxide (AAO) nanotemplates. *Advances in Natural Sciences: Nanoscience and Nanotechnology* **4**, 035013 (2013).

13. Dudem B, Ko YH, Leem JW, Lee SH, Yu JS. Highly transparent and flexible triboelectric nanogenerators with subwavelength-architectured polydimethylsiloxane by a nanoporous anodic aluminum oxide template. *ACS applied materials & interfaces* **7**, 20520-20529 (2015).

14. Hoa ND, Van Quy N, Cho Y, Kim D. An ammonia gas sensor based on non-catalytically synthesized carbon nanotubes on an anodic aluminum oxide template. *Sensors and Actuators B: Chemical* **127**, 447-454 (2007).

15. Zhou Q*, et al.* Tilted magnetic micropillars enabled dual-mode sensor for tactile/touchless perceptions. *Nano Energy* **78**, 105382 (2020).

16. Zhu P*, et al.* Skin-electrode iontronic interface for mechanosensing. *Nat Commun* **12**, 4731 (2021).

17. Lu P*, et al.* Iontronic pressure sensor with high sensitivity and linear response over a wide pressure range based on soft micropillared electrodes. *Science Bulletin* **66**, 1091-1100 (2021).

18. Bai N*, et al.* Graded Interlocks for Iontronic Pressure Sensors with High Sensitivity and High Linearity over a Broad Range. *ACS nano* **16**, 4338-4347 (2022).

19. Bai N*, et al.* Graded intrafillable architecture-based iontronic pressure sensor with ultra-broad-range high sensitivity. *Nat Commun* **11**, 209 (2020).

20. Sharma S*, et al.* Hydrogen-Bond-Triggered Hybrid Nanofibrous Membrane-Based Wearable Pressure Sensor with Ultrahigh Sensitivity over a Broad Pressure Range. *ACS nano* **15**, 4380-4393 (2021).

21. Xiao Y*, et al.* Multilayer double-sided microstructured flexible iontronic pressure sensor with a record-wide linear working range. *ACS sensors* **6**, 1785-1795 (2021).

22. Zheng Y*, et al.* Highly sensitive electronic skin with a linear response based on the strategy of controlling the contact area. *Nano Energy* **85**, 106013 (2021).

23. Gao L*, et al.* Highly sensitive pseudocapacitive iontronic pressure sensor with broad sensing range. *Nano-micro letters* **13**, 1-14 (2021).

24. Lin Q*, et al.* Highly sensitive flexible iontronic pressure sensor for fingertip pulse monitoring. *Advanced Healthcare Materials* **9**, 2001023 (2020).

25. Shen Z, Zhu X, Majidi C, Gu G. Cutaneous ionogel mechanoreceptors for soft machines, physiological sensing, and amputee prostheses. *Adv Mater* **33**, 2102069 (2021).

26. Zhou H*, et al.* Capacitive Pressure Sensors Containing Reliefs on Solution-Processable Hydrogel Electrodes. *ACS Applied Materials & Interfaces* **13**, 1441-1451 (2021).

27. Ruth SRA, Beker L, Tran H, Feig VR, Matsuhisa N, Bao Z. Rational design of capacitive pressure sensors based on pyramidal microstructures for specialized monitoring of biosignals. *Adv Funct Mater* **30**, 1903100 (2020).

28. Luo Z*, et al.* High-resolution and high-sensitivity flexible capacitive pressure sensors enhanced by a transferable electrode array and a micropillar–PVDF film. *ACS Applied Materials & Interfaces* **13**, 7635-7649 (2021).

29. Yang J*, et al.* Flexible, Tunable, and Ultrasensitive Capacitive Pressure Sensor with Microconformal Graphene Electrodes. *Acs Applied Materials & Interfaces* **11**, 14997-15006 (2019).

30. Guo Y, Gao S, Yue W, Zhang C, Li Y. Anodized aluminum oxide-assisted low-cost flexible capacitive pressure sensors based on double-sided nanopillars by a facile fabrication method. *ACS applied materials & interfaces* **11**, 48594-48603 (2019).

31. Niu H, Gao S, Yue W, Li Y, Zhou W, Liu H. Highly morphology-controllable and highly sensitive capacitive tactile sensor based on epidermis-dermis-inspired interlocked asymmetric-nanocone arrays for detection of tiny pressure. *Small* **16**, 1904774 (2020).

32. Qiu J*, et al.* Rapid-response, low detection limit, and high-sensitivity capacitive flexible tactile sensor based on three-dimensional porous dielectric layer for wearable electronic skin. *ACS applied materials & interfaces* **11**, 40716-40725 (2019).

33. Ha KH*, et al.* Highly Sensitive Capacitive Pressure Sensors over a Wide Pressure Range Enabled by the Hybrid Responses of a Highly Porous Nanocomposite. *Adv Mater* **33**, e2103320 (2021).

34. Xiong Y*, et al.* A flexible, ultra-highly sensitive and stable capacitive pressure

sensor with convex microarrays for motion and health monitoring. *Nano Energy* **70**, (2020).

35. Liu Y-Q, Zhang J-R, Han D-D, Zhang Y-L, Sun H-B. Versatile electronic skins with biomimetic micronanostructures fabricated using natural reed leaves as templates. *ACS applied materials & interfaces* **11**, 38084-38091 (2019).

36. Liu Z*, et al.* Natural bamboo leaves as dielectric layers for flexible capacitive pressure sensors with adjustable sensitivity and a broad detection range. *RSC Advances* **11**, 17291-17300 (2021).

37. Wan Y*, et al.* A highly sensitive flexible capacitive tactile sensor with sparse and high-aspect-ratio microstructures. *Advanced Electronic Materials* **4**, 1700586 (2018).

38. Quan Y*, et al.* Highly sensitive and stable flexible pressure sensors with micro-structured electrodes. *J Alloy Compd* **699**, 824-831 (2017).

39. Wan Y*, et al.* Natural plant materials as dielectric layer for highly sensitive flexible electronic skin. *Small* **14**, 1801657 (2018).

40. Lee K*, et al.* Rough-surface-enabled capacitive pressure sensors with 3D touch capability. *Small* **13**, 1700368 (2017).

41. Zhu B*, et al.* Hierarchically Structured Vertical Gold Nanowire Array-Based Wearable Pressure Sensors for Wireless Health Monitoring. *ACS Appl Mater Interfaces* **11**, 29014-29021 (2019).

42. Yang JC*, et al.* Microstructured Porous Pyramid-Based Ultrahigh Sensitive Pressure Sensor Insensitive to Strain and Temperature. *ACS Appl Mater Interfaces* **11**, 19472-19480 (2019).

43. Mannsfeld SC*, et al.* Highly sensitive flexible pressure sensors with microstructured rubber dielectric layers. *Nature materials* **9**, 859-864 (2010).

44. Cho SH*, et al.* Micropatterned pyramidal ionic gels for sensing broad-range pressures with high sensitivity. *ACS applied materials & interfaces* **9**, 10128-10135 (2017).

45. Qiu Y*, et al.* Bioinspired, multifunctional dual-mode pressure sensors as electronic skin for decoding complex loading processes and human motions. *Nano Energy* **78**, 105337 (2020).

46. Luo Y*, et al.* Flexible Capacitive Pressure Sensor Enhanced by Tilted

Micropillar Arrays. *ACS Appl Mater Interfaces* **11**, 17796-17803 (2019).

47. Sharma S, Chhetry A, Sharifuzzaman M, Yoon H, Park JY. Wearable capacitive pressure sensor based on MXene composite nanofibrous scaffolds for reliable human physiological signal acquisition. *ACS applied materials & interfaces* **12**, 22212-22224 (2020).

48. Chhetry A, Sharma S, Yoon H, Ko S, Park JY. Enhanced sensitivity of capacitive pressure and strain sensor based on CaCu3Ti4O12 wrapped hybrid sponge for wearable applications. *Adv Funct Mater* **30**, 1910020 (2020).

49. Tay RY*, et al.* Lightweight, superelastic boron nitride/polydimethylsiloxane foam as air dielectric substitute for multifunctional capacitive sensor applications. *Adv Funct Mater* **30**, 1909604 (2020).

50. Jin T*, et al.* Ultrathin nanofibrous membranes containing insulating microbeads for highly sensitive flexible pressure sensors. *ACS applied materials & interfaces* **12**, 13348-13359 (2020).

51. Liu Q*, et al.* Highly transparent and flexible iontronic pressure sensors based on an opaque to transparent transition. *Advanced Science* **7**, 2000348 (2020).

52. Choi J*, et al.* Synergetic effect of porous elastomer and percolation of carbon nanotube filler toward high performance capacitive pressure sensors. *ACS applied materials & interfaces* **12**, 1698-1706 (2019).

53. Yu P*, et al.* All-Fabric Ultrathin Capacitive Sensor with High Pressure Sensitivity and Broad Detection Range for Electronic Skin. *ACS Applied Materials & Interfaces*, (2021).
