# Supplementary material for: Iontronic pressure sensor with high sensitivity over ultra-broad linear range enabled by laser-induced gradient micro-pyramids
Source: Nat Commun. 2023 Jun 1;14:2907. doi: 10.1038/s41467-023-38274-2 (PMC10235028; doi:10.1038/s41467-023-38274-2)
Supplement: Supplementary file 3 — Description of Additional Supplementary Files [file 41467_2023_38274_MOESM3_ESM.pdf]

Description of Additional Supplementary Files

File Name: Supplementary Movie 1
Description: The mechanical simulation displaying the stress distribution of the different microstructures under pressure loading.

File Name: Supplementary Movie 2
Description: The response time measured by loading a 50g weight.

File Name: Supplementary Movie 3
Description: The response time measured by using a linear actuator.

File Name: Supplementary Movie 4
Description: The experiment to measure the limit of detection.

File Name: Supplementary Movie 5
Description: The experiment to measure the pressure resolution.

File Name: Supplementary Movie 6
Description: Interaction with the robotic hand.
