# Supplementary figures and images for: Iontronic pressure sensor with high sensitivity over ultra-broad linear range enabled by laser-induced gradient micro-pyramids
Source: Nat Commun. 2023 Jun 1;14:2907. doi: 10.1038/s41467-023-38274-2 (PMC10235028; doi:10.1038/s41467-023-38274-2)

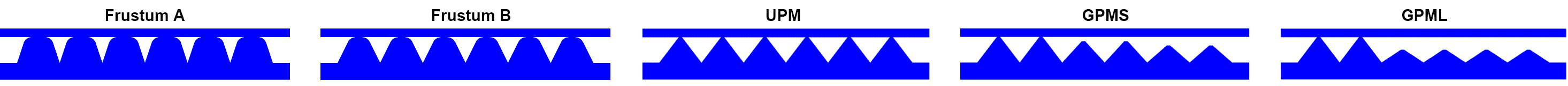

Supplement: Supplementary file 4 — Supplementary Movie 1 [file 41467_2023_38274_MOESM4_ESM.gif]
