# Supplementary material for: Iontronic pressure sensor with high sensitivity over ultra-broad linear range enabled by laser-induced gradient micro-pyramids
Source: Nat Commun. 2023 Jun 1;14:2907. doi: 10.1038/s41467-023-38274-2 (PMC10235028; doi:10.1038/s41467-023-38274-2)
Supplement: Supplementary file 10 — Reporting Summary [file 41467_2023_38274_MOESM10_ESM.pdf]

nature research

Corresponding author(s): Huanyu Cheng

Lasing Reporting Summary

Nature Research wishes to improve the reproducibility of the work that we publish. This form is intended for publication with all accepted papers reporting claims of lasing and provides structure for consistency and transparency in reporting. Some list items might not apply to an individual manuscript, but all fields must be completed for clarity.

For further information on Nature Research policies, including our data availability policy, see Authors & Referees.

Experimental design

Please check: are the following details reported in the manuscript?

1. Threshold

Plots of device output power versus pump power over a wide range of values indicating a clear threshold

- [ ] Yes
- [x] No

Not necessary for the fabrication of the microstructure template, with the universal laser system (VLS 2.30) operated far above the threshold.

2. Linewidth narrowing

Plots of spectral power density for the emission at pump powers below, around, and above the lasing threshold, indicating a clear linewidth narrowing at threshold

- [ ] Yes
- [x] No

Not necessary for the fabrication of the microstructure template.

Resolution of the spectrometer used to make spectral measurements

- [ ] Yes
- [x] No

Spectral measurements were not performed.

3. Coherent emission

Measurements of the coherence and/or polarization of the emission

- [ ] Yes
- [x] No

Not necessary for the fabrication of the microstructure template.

4. Beam spatial profile

Image and/or measurement of the spatial shape and profile of the emission, showing a well-defined beam above threshold

- [ ] Yes
- [x] No

Not necessary for the fabrication of the microstructure template.

5. Operating conditions

Description of the laser and pumping conditions
Continuous-wave, pulsed, temperature of operation

- [x] Yes
- [ ] No

"Fabrication of the PMMA mold" Section and Supplementary Table 1.

Threshold values provided as density values (e.g. W cm^{-2} or J cm^{-2}) taking into account the area of the device

- [ ] Yes
- [x] No

Not necessary for the fabrication of the microstructure template.

6. Alternative explanations

Reasoning as to why alternative explanations have been ruled out as responsible for the emission characteristics
e.g. amplified spontaneous, directional scattering; modification of fluorescence spectrum by the cavity

- [ ] Yes
- [x] No

Not necessary for the fabrication of the microstructure template.

7. Theoretical analysis

Theoretical analysis that ensures that the experimental values measured are realistic and reasonable
e.g. laser threshold, linewidth, cavity gain-loss, efficiency

- [ ] Yes
- [x] No

Not necessary for the fabrication of the microstructure template.

8. Statistics

Number of devices fabricated and tested

- [ ] Yes
- [x] No

Not necessary for the fabrication of the microstructure template.

Statistical analysis of the device performance and lifetime (time to failure)

- [ ] Yes
- [x] No

There was no statistical analysis on this device.
